# Quantitative Phylogenomics of Within-Species Mitogenome Variation: Monte Carlo and Non-Parametric Analysis of Phylogeographic Structure among Discrete Transatlantic Breeding Areas of Harp Seals (*Pagophilus groenlandicus*)

**DOI:** 10.1371/journal.pone.0134207

**Published:** 2015-08-24

**Authors:** Steven M. Carr, Ana T. Duggan, Garry B. Stenson, H. Dawn Marshall

**Affiliations:** 1 Genetics, Evolution, and Molecular Systematics Laboratory, Department of Biology, Memorial University of Newfoundland, St John's, NL A1B 3X9, Canada; 2 Wildlife Genetics and Genomics Laboratory, Department of Biology, Memorial University of Newfoundland, St John's, NL A1B 3X9, Canada; 3 Marine Mammals Section, Science Branch, Dept. of Fisheries and Oceans, PO Box 5667, St. John's, Nfld., A1C 5X1, Canada; BiK-F Biodiversity and Climate Research Center, GERMANY

## Abstract

Phylogenomic analysis of highly-resolved intraspecific phylogenies obtained from complete mitochondrial DNA genomes has had great success in clarifying relationships within and among human populations, but has found limited application in other wild species. Analytical challenges include assessment of random versus non-random phylogeographic distributions, and quantification of differences in tree topologies among populations. Harp Seals (*Pagophilus groenlandicus* Erxleben, 1777) have a biogeographic distribution based on four discrete trans-Atlantic breeding and whelping populations located on “fast ice” attached to land in the White Sea, Greenland Sea, the Labrador ice Front, and Southern Gulf of St Lawrence. This East to West distribution provides a set of *a priori* phylogeographic hypotheses. Outstanding biogeographic questions include the degree of genetic distinctiveness among these populations, in particular between the Greenland Sea and White Sea grounds. We obtained complete coding-region DNA sequences (15,825 bp) for 53 seals. Each seal has a unique mtDNA genome sequence, which differ by 6 ~ 107 substitutions. Six major clades / groups are detectable by parsimony, neighbor-joining, and Bayesian methods, all of which are found in breeding populations on either side of the Atlantic. The species coalescent is at 180 KYA; the most recent clade, which accounts for 66% of the diversity, reflects an expansion during the mid-Wisconsinan glaciation 40 ~ 60 KYA. F_ST_ is significant only between the White Sea and Greenland Sea or Ice Front populations. Hierarchal AMOVA of 2-, 3-, or 4-island models identifies small but significant Φ_SC_ among populations within groups, but not among groups. A novel Monte-Carlo simulation indicates that the observed distribution of individuals within breeding populations over the phylogenetic tree requires significantly fewer dispersal events than random expectation, consistent with island or *a priori* East to West 2- or 3-stepping-stone biogeographic models, but not a simple 1-step trans-Atlantic model. Plots of the cumulative pairwise sequence difference curves among seals in each of the four populations provide continuous proxies for phylogenetic diversification within each. Non-parametric Kolmogorov-Smirnov (K-S) tests of maximum pairwise differences between these curves indicates that the Greenland Sea population has a markedly younger phylogenetic structure than either the White Sea population or the two Northwest Atlantic populations, which are of intermediate age and homogeneous structure. The Monte Carlo and K-S assessments provide sensitive quantitative tests of within-species mitogenomic phylogeography. This is the first study to indicate that the White Sea and Greenland Sea populations have different population genetic histories. The analysis supports the hypothesis that Harp Seals comprises three genetically distinguishable breeding populations, in the White Sea, Greenland Sea, and Northwest Atlantic. Implications for an ice-dependent species during ongoing climate change are discussed.

## Introduction

Analyses of multiple complete intraspecific mtDNA genomes were first applied to humans to clarify the historical emergence of modern humans and their subsequent migrations Out of Africa [1]. The accumulation of many thousands of such genomes has clarified more recent population movements in great detail, including those influenced by successive Holocene glaciations [2]. For example, high-resolution mitogenomic sampling of Iberian refugial lineages effectively discriminates postglacial dispersal and ecological events [3]. Only a few other wild species have been the subject of extensive mitogenomic phylogeographic analysis. Atlantic Cod (*Gadus morhua*) have a much more ancient species structure than previously suspected, with coalescence during the Wisconsinan glacial, and major clade differentiation at the peak of the Sangamon / Würm interglacial rather than subsequent to the last Recent glacial [4]. The major clades are phylogeographically mixed, and well-separated geographic samples coalesce only towards the base of the gene tree. The sister species of Atlantic Cod, the Walleye Pollock (*Gadus chalcogrammus*), includes geographically isolated populations originally supposed to be of recent origin that are shown by mitogenomic analysis to be ancient [5]. In contrast, separate geographic populations of freshwater whitefish (*Coregonus*) are discrete genomic clades [6]. Major phylogenetic lineages in Killer Whales (*Orcinus*) correspond to discrete geographic populations including some assignable to subspecies [7], and in Fin Whales (*Balaenoptera*) to what appear to be recognizable species [8].

Standard methods of phylogenetic analysis and (or) quantitative assessment of within-species mtDNA population structure founder when every individual is a distinct branch. Hierarchal analysis of molecular variance (AMOVA) among haplotypes *per se* is uninformative, as the entire variance occurs among individuals with respect to the total (F_ST_ = 1.0). AMOVA based on nucleotide divergences between haplotypes (ϕ_ST_ < 1.0) may partition variance among populations (ϕ_SC_ > 0.0,) or groups (ϕ_CT_ > 0.0), but does not capture phylogenetic structure within and among populations. Similarly, row by column tests of the relative abundances of major clades across populations cannot take into account phylogenetic relationships either within or among those clades [9]. Evaluation of hypotheses that phylogeographic subcomponents within species have different phylogenetic tree topologies is elusive, not least because it is difficult to define precisely what may be meant by ‘different’. Simple phylogenetic clustering [7,8] or nested clade analysis is informative for more overt structures wherein discrete clades are nested within others [10], but NCA and other coalescence analyses are less informative when geographic population components are of mixed genetic composition and clades coalesce only at the most basal levels [4]. We require novel methods that can be tested on species with phylogeographies that provide *a priori* hypotheses.

**Fig 1:**
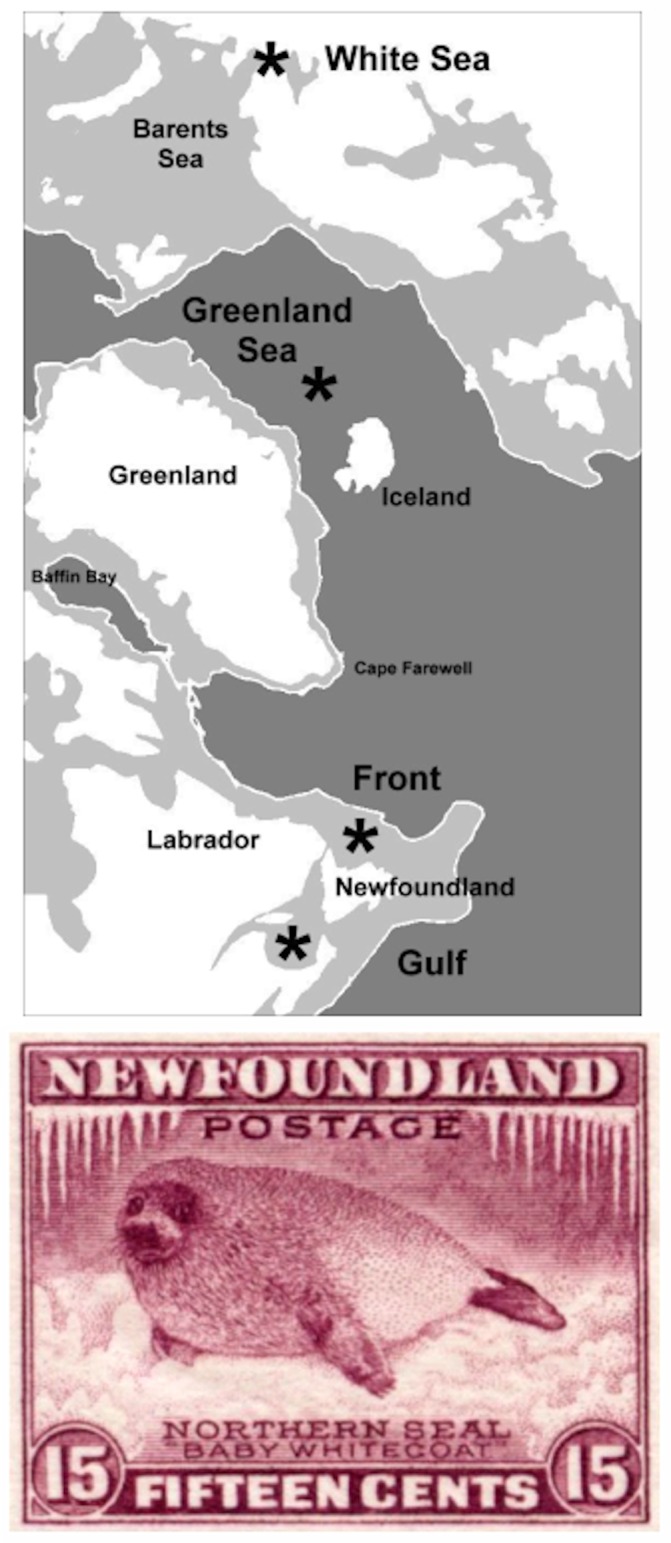
Breeding and whelping areas of Harp Seals (*Pagophilus groenlandicus*) and the origins of samples used in this study (from [22]). The Dominion of Newfoundland was a separate nation until union with Canada in 1949, and its postage stamps often depicted wildlife, including Harp Seals. The stamp shows a “White Coat”, a neonatal seal prior to weaning at ca. 12 days of age.

Harp seals (*Pagophilus groenlandicus* Erxleben 1777) are endemic to the North Atlantic and adjacent Arctic waters (Fig 1), where they are the most abundant pinniped [11]. The genus name refers to their “ice-loving” habit, and their whelping (pupping) and molting are associated with drifting pack ice. The species comprises exactly four populations associated with discrete whelping and breeding areas in the White Sea, in the Greenland Sea near Jan Mayen Island, and in the Northwest Atlantic along the east coast of Canada. The latter has been separated into two major sub-areas, one on the pack ice off the coast of northeast Newfoundland and southern Labrador and the other in the southern Gulf of St. Lawrence. During the whelping period, seals in each area may form one or several concentrations (“patches”), which may be split, mixed, and reformed by current- and wind-driven ice movements. There is an extremely rapid, two-week nursing period, and breeding occurs immediately after the pups are weaned. Harp seals undertake extensive migrations between winter whelping areas and summer feeding areas, and the ranges of adult and immature seals from different populations are known to overlap during the non-breeding season [11,12,13,14,15]. Animals are exchanged between the Gulf and Front populations through the Strait of Belle Isle between the Northern Peninsula of Newfoundland and the Quebec mainland. Exchange between the White and Greenland Seas and the Northwest Atlantic occurs along pack ice attached to the Norwegian and southern Greenland coasts, and through the Davis Strait between Greenland and Canada. Tag return data indicate a high degree of breeding site fidelity of adults, although there are rare reports of pups tagged in one whelping area, and recovered as adults from other areas shortly after the breeding period [11,12,13,16,17,18,19,20,21]. It is therefore possible that there are genetically effective exchanges of reproductive adults among breeding areas.

Relationships among the four populations have been examined by means of cranial measurements, underwater vocalizations, and a variety of genetic methods including allozyme and minisatellite analysis (reviewed in [22]). Collectively, these studies suggest that the Northwest Atlantic breeding areas are to some degree reproductively isolated from the Greenland Sea and White Sea populations, but there have been no clear indications of genetic differentiation between the latter two populations. Microsatellite studies of other fast-ice seal species have not identified significant population structure [23], including Hooded Seals (*Cystophora cristata*) at Jan Mayen Island *versus* those in the Northwest Atlantic [24].

Besides a conventional AMOVA, we introduce here two novel methods for analysis of intraspecific gene trees, as applied to an examination of the mitogenomic structure of Harp Seal breeding areas. These are a Monte Carlo simulation of phylogeographic distribution, and a non-parametric analysis of cumulative pairwise mismatch distributions between populations. The discrete, ordered geographic distribution of the Harp Seal breeding and whelping areas provides several *a priori* hypotheses of phylogeographic relationships. With mitogenomic data that distinguish each individual as a discrete “twig” in the intraspecific family tree, it is possible to evaluate these hypotheses quantitatively. The results show that fine distinctions among populations can be made with mitogenomic data, which with respect to AMOVA methods offer an improved view of relationships among breeding areas that bears on biological and ecological questions of genetic connectivity among populations.

## Results

Each of the 53 seals had a unique 15,825 bp genome sequence, which differed by 7 to 106 substitutions (mean = 50.9 ± 19.8) among 588 variable sites (S1 Table). We designate the southern Gulf, Newfoundland and Labrador ice front, Greenland Sea, and White Sea population samples as SG, NL, GS, and WS, respectively, and the Northwest and Northeast Atlantic sample population pairs as NWA and NEA, respectively. The sequences have been submitted to GenBank and assigned the accession numbers KP942529—KP942581.

### Topological structure

**Fig 2:**
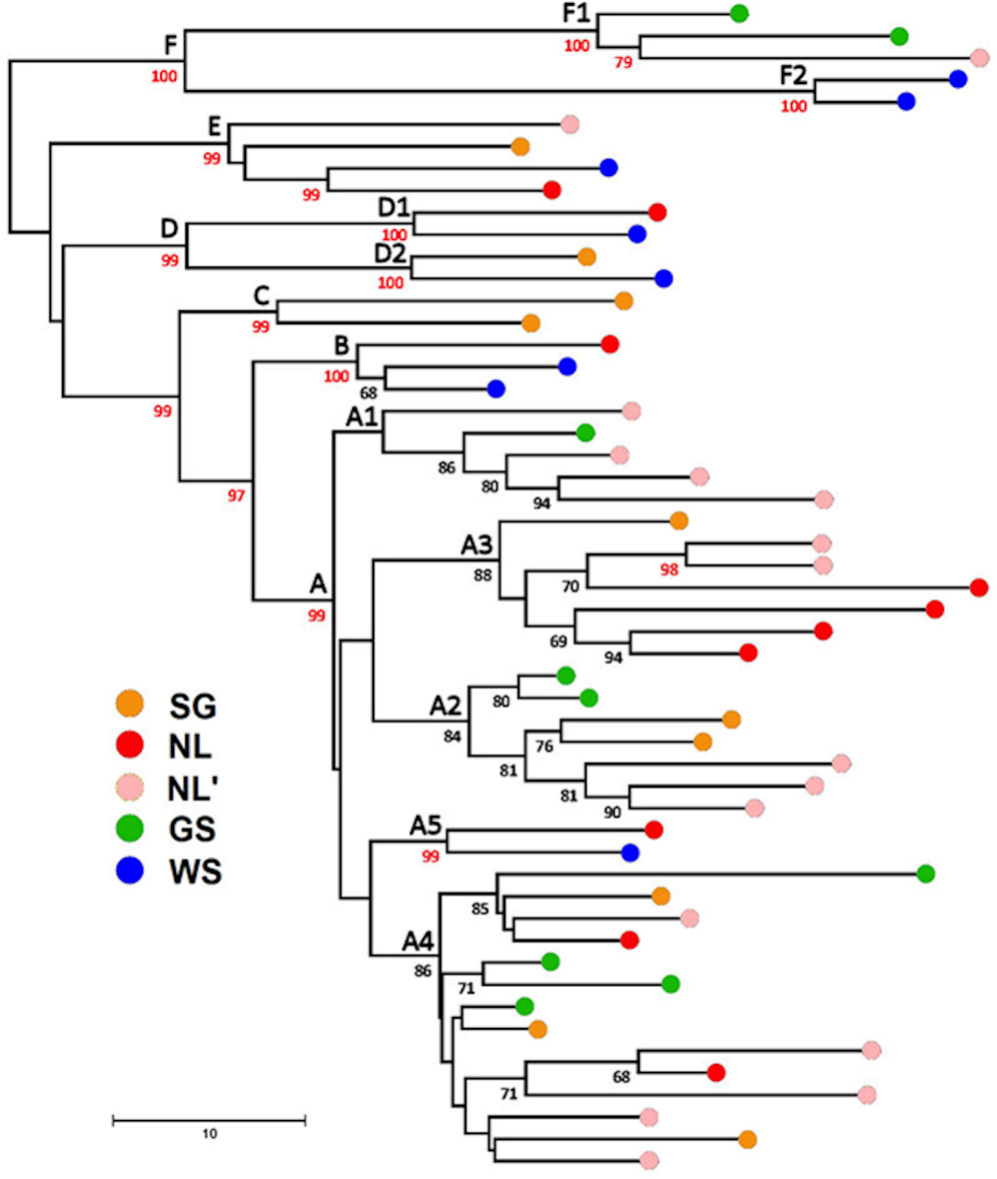
Mitochondrial DNA genome phylogeography of 53 Harp Seals. Neighbor-joining tree rooted with respect to the next most closely related species, the Ribbon Seal (*Histriophoca fasciata*) [25]. Clusters supported by >67% of 10,000 bootstrap replications are indicated by numbers below the corresponding vertex; support >95% is indicated in red. Origins of samples from the four breeding areas (including separate Gulf and Front samples) are indicated by colored circles. The six primary groups / clades and their inter-relationships ([**F**+ [[**E** + **D**] + [**C** + [**B**+**A**]]]]) are all supported by >97% of NJ and MP bootstraps.

**Fig 3:**
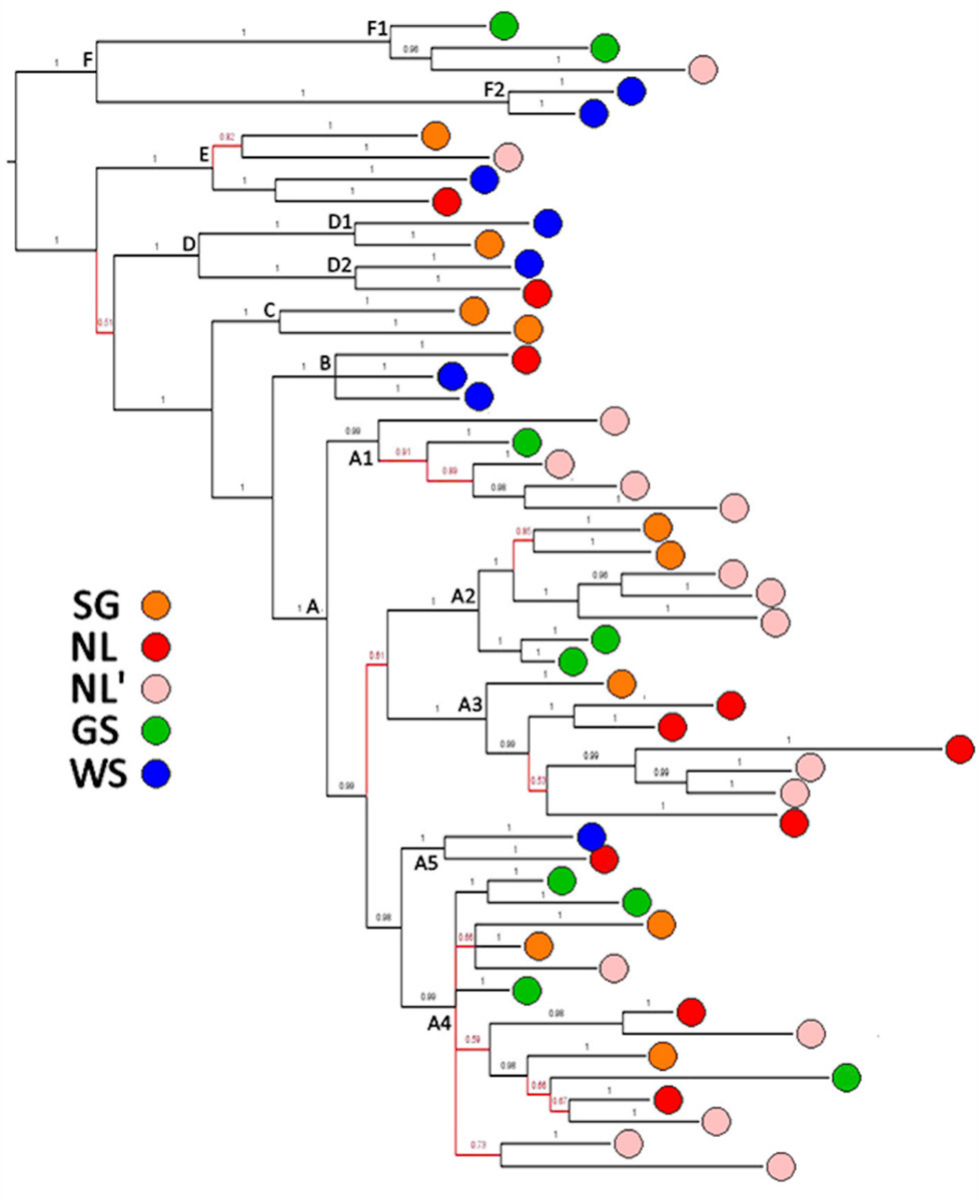
Bayes analysis of mtDNA genome phylogeography of Harp Seals. Tree rooted as in Fig 2. Nodes supported by posterior probabilities of p ≥ 0.95 are shown in black, those supported by 0.50 ≤ p < 0.95 are shown in red. All groups identified by > 95% of bootstraps in Fig 2 are supported with posterior probabilities p ~ 1.00, as are relationships within A. Differences in relationships within subgroup A4 are not strongly differentiated by bootstrap or posterior probabilities.

**Fig 4:**
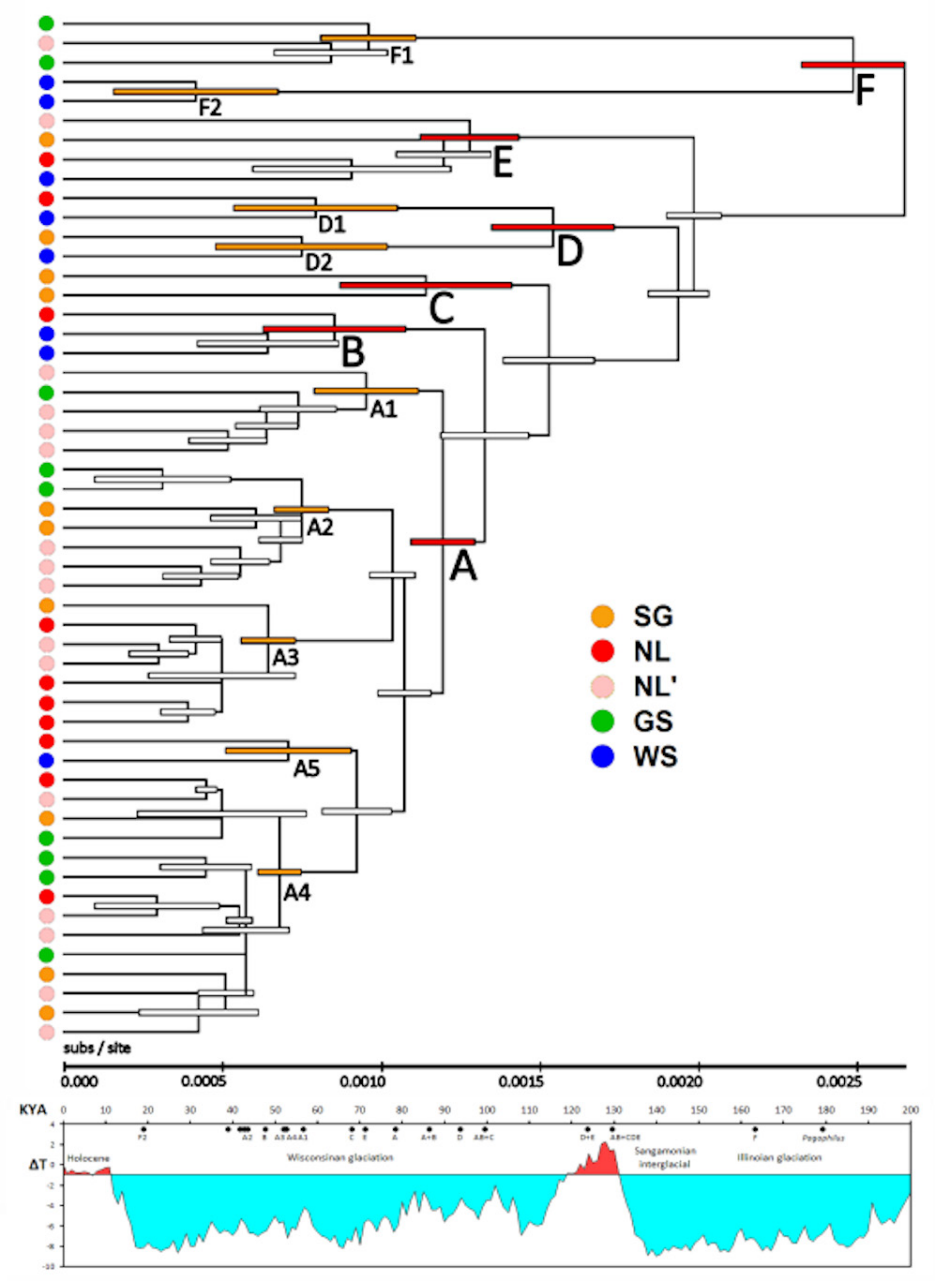
Linearized maximum likelihood tree for mtDNA genome phylogeography of Harp Seals. (a) Rates of substitution per site are estimated from the clock model in Fig 5A; confidence intervals (±95%) are indicated by bars. (b) Correlation of Holarctic temperature and glacial histories with times of clade origins. The temperature trend over the last 200 Kyr is taken from the Vostok ice core data, which are tied to analogous data from Greenland [4].

Bootstrap analysis of the neighbor-joining tree identified six primary groups (designated **A**, **B**, **C**, **D**, **E**, and **F**) and a further nine sub-groups or pairs within these at ≥95% (Fig 2; Table 1). Within the majority **A** group (66% of all seals), five subgroups designated **A1**-**A5** are supported in 84 ~ 99% of replicates, as are two subgroups each in **D** and **E**. Parsimony analysis based on 243 phylogenetically informative sites identified n = 57 minimum-length trees of length L = 808, and bootstrap analysis identified the same 34 groups in the NJ analysis as clades, including the six primary groups in 87 ~ 99% of replicates (results not shown). Unresolved relationships in the topology of the phenetic and cladistic trees occur only within the **A** clade, in particular basal relationships within **A3** and **A4** and the relative branching order of **A2** and **A3** with respect to **A4** / **A5**. In general, NJ relationships supported in <67% are unresolved in the strict-consensus of the 57 L = 808 MP trees. A Bayes analysis also produces the same topology, with posterior probabilities > 0.95 supporting the nodes above, as well as among designated subgroups within clade **A** (Fig 3). Relationships within **A4** remain unresolved and by the method collapse as a polytomy. A maximum likelihood tree based on a clock-constrained general linear model (see below) again identifies the same topology as the NJ and MP analyses, with nodes **A-F** and **A1-A5** all supported at >95%, again with unresolved relationships only within **A4** (Fig 4). With whole-mitogenome sequences, identification of well-supported groups is largely method-independent. The coincident groups identified by NJ, ML, MP, and Bayes analysis are hereinafter referred to as ‘clades’.

**Table 1:** Summary of genetic diversity within major groups and clades of Harp Seals.

Clade	Mean	s.d.	Min	Max	N
**F**	55.0	27.3	13	82	5
**E**	33.3	4.1	27	39	4
**D**	39.7	12.3	23	51	4
**C**	32.0	-	-	-	2
**B**	20.7	4.2	16	224	2
**A**	36.3	10.1	7	65	35
**A1**	23.8	6.1	14	35	5
**A2**	22.2	5.3	7	29	7
**A3**	30.7	7.7	15	49	7
**A4**	25.4	8.4	8	49	2
**A5**	36.6	-	-	-	14
**Total**	51.6	19.8	7	106	53

### Molecular clock and population history

**Fig 5:**
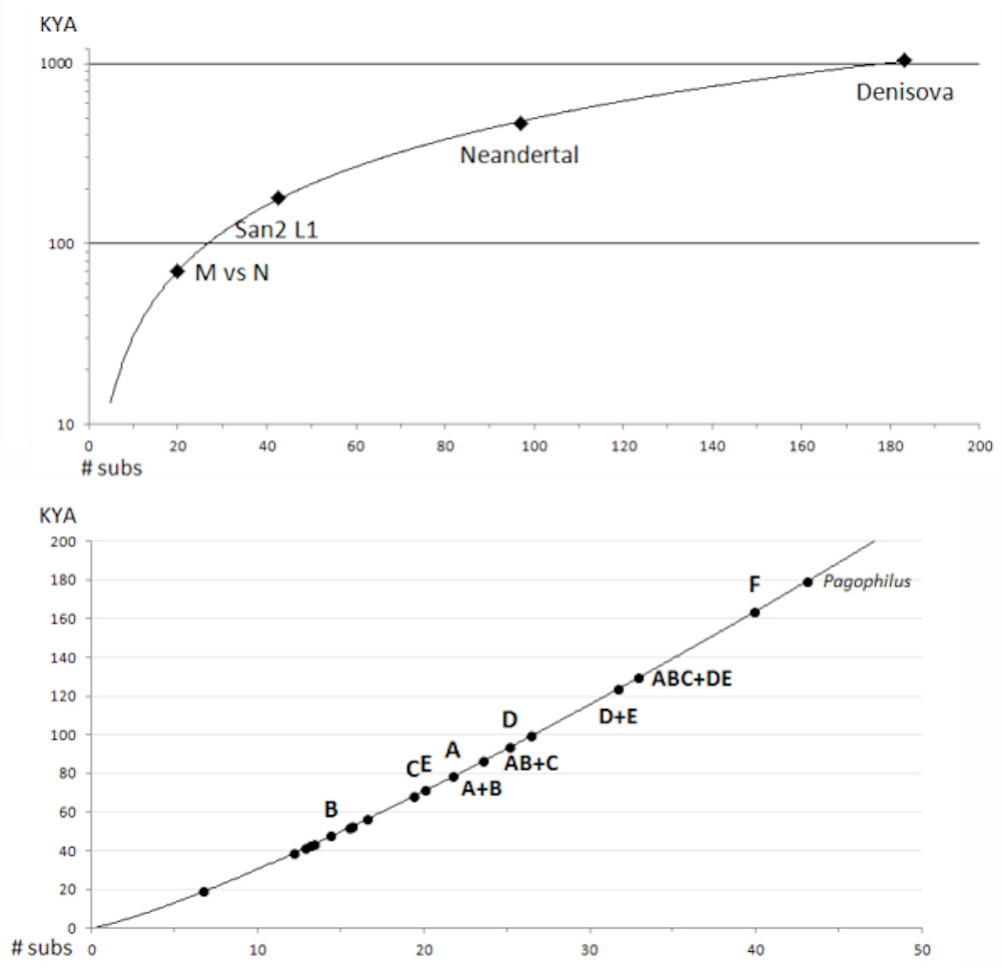
Calibration of intra-specific divergences among Harp Seal clades. (a) ML clock-corrected estimates of the molecular distances to the MRCA for four hominines with established divergence dates < 1 MYA follow a power curve relationship T = 1.886 x (d)^1.21^ (r^2^ = 0.9983), where T = KYA and d = linearized ML substitution rates (semi-log plot). Sources of homine mtDNA genomes are given in Methods: San2 is in the L1 haplogroup that is the basal divergence from other extant *Homo* [1], M vs N is the divergence between haplotypes in the U and A haplogroups, respectively [9]. (b) Dates of the MRCA of the major intra-specific *Pagophilus* clades estimated from the same power curve (linear plot).

Fig 5 shows a power curve fit of the ML-corrected substitutions for the MRCA of the four hominines to the reference mitogenome, with the equation T = 1.886 x (d)^1.21^ (r^2^ = 0.9983), where T = time in KY and d = ML-corrected substitutions (Fig 5A). Use of the same curve to estimate intra-specific *Pagophilus* coalescents gave the results shown in Fig 5B and as compared to glacial history in Fig 4. The species ancestral node [**ABCDE + F**] occurs in the middle of the Illinoian / Saale glaciation ~180 KYA. The ancestral node of the **AB+C** and **ABC+ DE** superclades occur during the Sangamonian interglacial 120 ~ 130 KYA. The ancestral node of Clade **A**, which comprises 66% of genomes examined, occurs at ~80 KYA, and its five component subclades alongside three of the four in **D** and **F** coalesce ca. 40 ~ 60 MYA during the mid-Wisconsinan.

**Fig 6:**
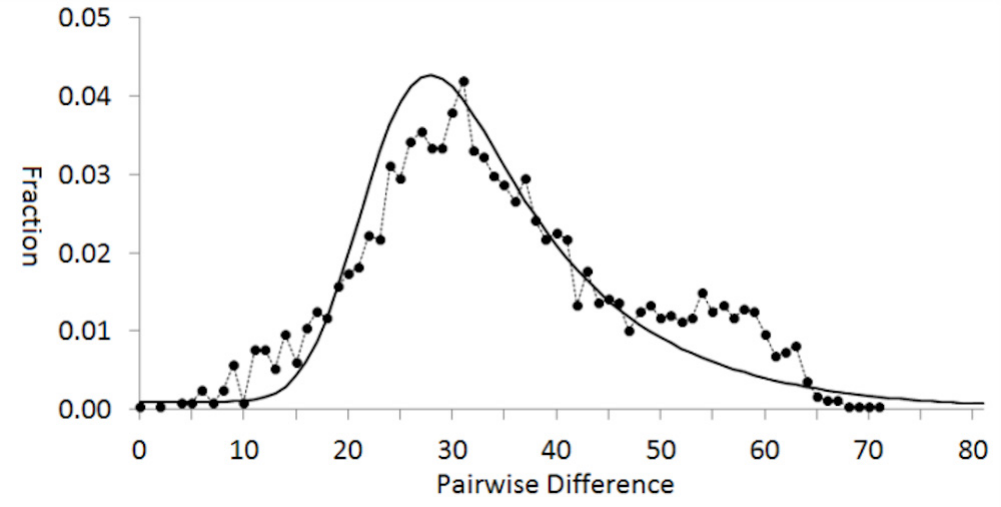
Population expansion as inferred from the pairwise mismatch distribution of Harp Seals (DnaSP: [54]). The null hypothesis of constant population size is rejected (Fu & Li’s [26] and Tajima’s [27] D statistics both p << 0.01) in favor of a population expansion with an estimated τ = 33.4 corresponding to the upward inflection of the peak, at 57 KYA by the calibration in Fig 4.

Analysis of the pairwise mismatch distribution rejects the hypothesis of constant population size (Fu & Li’s [26] and Tajima’s [27] D statistics both p << 0.01) in favor of a population expansion at 53 KYA, as estimated from τ = 37.4 (Fig 6). This coincides with the origin and diversification of subclades within the modal clade **A**, which includes 66% of the samples.

### Analysis of molecular variance

Because every seal has a distinct haplotype sequence, hierarchal AMOVA based on haplotypic diversity *per se* assigns the entire variance as among individuals, rather than to any among-population-within-group or among-group structure (F_ST_ = 1.00, F_SC_ = F_CT_ = 0.00). We calculated AMOVAs based on nucleotide distances (ϕ statistics) among haplotypes for all pairwise combinations of breeding grounds (Table 2), and for four partitions of breeding grounds based on three *a priori* and one *a posteriori* biogeographic models (Table 3). These are (I) a non-hierarchal four-island model [without an among-group ϕ component], (II) a two-island Trans-Atlantic model with groups NWA and NEA, (III) a three-island model with group NWA, and (IV) based on Table 2, a two-island model with WS contrasted with the other three breeding grounds grouped. In all models, 0.88 ~ 0.94 of the variance occurs among haplotypes within populations (p << 0.01) as expected. Variance among populations within groups ϕ_SC_ [= ϕ_ST_ in Model I] is small (0.02 ~ 0.06) but significant (p <0.05), except in Model IV. None of the hierarchal Models II ~ IV has a significant among-group ϕ_CT_. Among-group variance components are dominated by the relative distinctness of WS, but even in Model IV with ϕ_CT_ > 0.10 this does not approach significance (p > 0.25).

**Table 2:** AMOVA results. Pairwise ϕ_ST_ values for the four breeding grounds [lower triangular matrix] and their significance values [upper triangular matrix]. Abbreviations: GS = southern Gulf, NL = Newfoundland and Labrador ice front, GS = Greenland Sea, WS = White Sea, NWA = GS and NL, NEA = GS and WS.

	SG	NL	GS	WS
**SG**	-	0.11761	0.41441	0.05871
**NL**	0.02007	-	0.09019	*0*.*00089* **
**GS**	0.00357	0.02775	-	*0*.*03366* *
**WS**	0.05799	0.12979	0.09921	-

* = p < 0.05

** = p < 0.01.

**Table 3:** AMOVA within and among breeding grounds of Harp Seals: alternative island models. Four models were evaluated (I-IV), as described in Methods.

Model Groups		Source	df	Variance component	% variance	Fixation indices		*p*	* *
I	Four-Island:	*among groups*	-	-	-	* *			
	SG NL GS WS	among pops	3	1.5303	5.80	*F*_*ST*_	0.0580	0.0020	**
		w/i pops	49	24.8392	94.20	* *			
									
II	Trans-Atlantic:	among groups	1	0.1502	0.57	F_CT_	0.0057	0.6721	
** **	(SG NL) (GS WS)	among pops w/i groups	2	1.4330	5.42	F_SC_	0.0545	0.0188	*
** **	** **	w/i pops	49	24.8392	94.01	F_ST_	0.0599	0.0022	**
									
III	Three-Island:	among groups	2	1.2170	4.56	F_CT_	0.0456	0.2994	
	(SG NL) GS WS	among pops w/i groups	2	0.9471	3.55	F_SC_	0.0372	0.0133	*
		w/i pops	48	24.5247	91.89	F_ST_	0.0811	0.0010	**
									
IV	Two-Island:	among groups	1	2.8901	10.26	F_CT_	0.1026	0.2575	
	(SG NL GS) WS	among pops w/i groups	2	0.4282	1.52	F_SC_	0.0170	0.0880	
		w/i pops	49	24.8392	88.22	F_ST_	0.1179	0.0020	**

* = p < 0.05

** = p < 0.01.

### Quantitative phylogenomics: Monte Carlo and non-parametric methods

**Fig 7:**
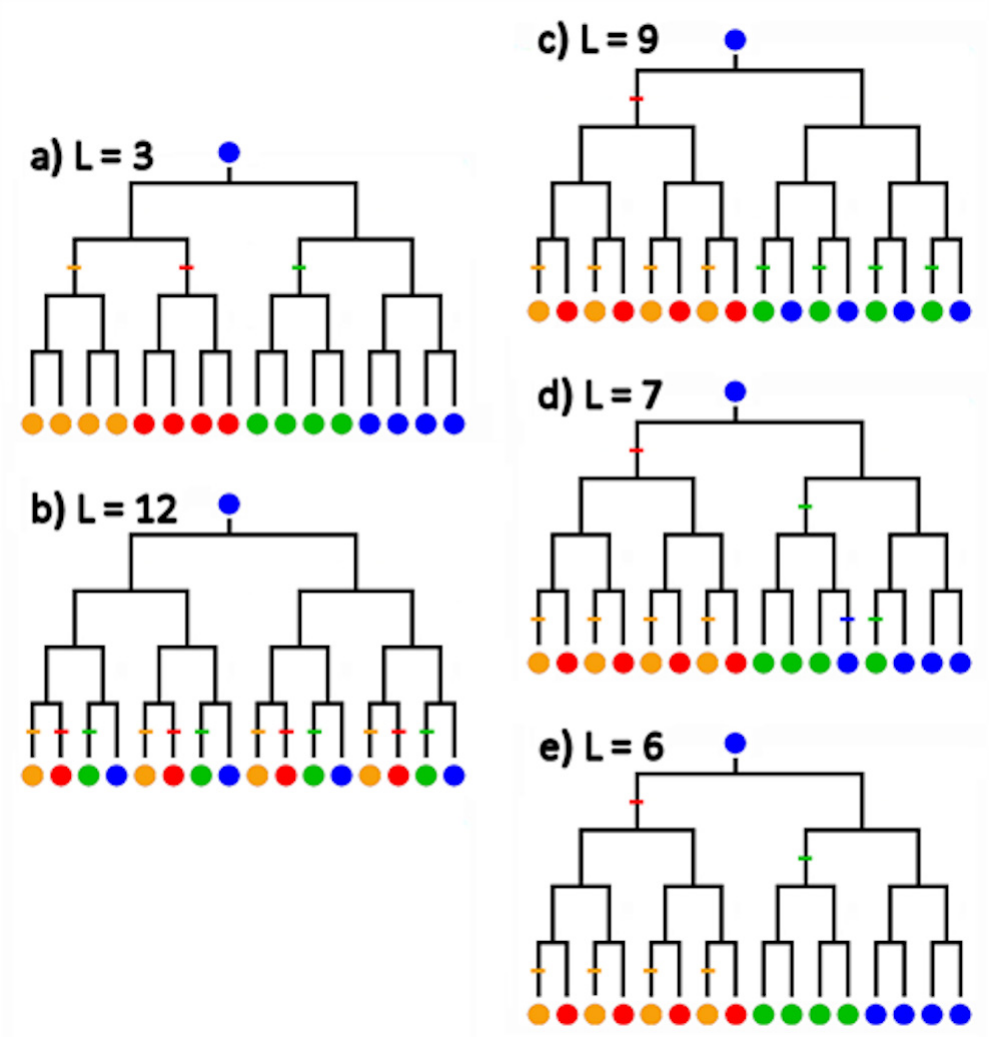
Dispersal tree length decreases as phylogeography becomes more structured. Consider a dichotomously-branching mitogenomic tree of 16 individuals distributed among four geographic areas (red, orange, green, and blue), with one population (blue) designated as ancestral. (A) The most highly structured model, in which each of the three alternative areas corresponds to a separate clade originating by a single dispersal event, requires just L = 3 events. (B) The least structured model, in which clades are distributed uniformly among areas such that there is no correspondence between genetic relationship and population, requires L = 12 events. [Model drawn with delayed events, and terminal red / orange and green / blue pairs: alternative event distributions are possible]. Intermediate structures include: (C) Separate clades in the red and orange areas and in the green and blue areas, but uniform geographic distribution within those two clades (L = 9), (D) as in (C) but with minimal exchange between blue and green areas (L = 7), and (E) as in (C) but with complete separation of blue and green (L = 6). The models are drawn with delayed dispersal events: other event distributions are possible. Models C, D, & E are analogous respectively to a two-population Harp Seal model, a three-population mixed model with a uniform single Northwest Atlantic population and limited exchange between the Greenland and White Seas, and a pure three-population model with separate Greenland and White Sea populations (cf. Fig 8).

Because the hierarchal groups within AMOVA models are intrinsically unordered, they can capture island models but not linear stepping-stone models based on *a priori* hypotheses corresponding to the east-to-west arrangement of Harp Seal breeding grounds. Where the AMOVA is based on pairwise distances, it also cannot capture phylogenetic differences among breeding groups in their distributions as haplotypes across a gene tree topology. We therefore developed a novel Monte Carlo analysis that accommodates island and stepping-stone models, as well as topological heterogeneity among groups. The logic of our approach is illustrated in Fig 7: the more highly structured a species is with respect to clade distribution among breeding areas, the smaller the number of dispersal events required to explain an observed distribution. The null hypothesis of an unstructured species may then be tested by comparing, for the topology of an observed tree (Fig 2), the length L of the observed distribution of breeding area assignments with the random distribution of L for assignments as generated by Monte Carlo simulation, for any phylogeographic model. The null hypothesis of no structure is rejected when the observed L falls in the lower (one-tailed) 5% of the random distribution. The smaller the observed L and the smaller the area of the tail in which it occurs, the greater the departure from the model-specific random distribution, and the stronger the structure. For example, in Harp Seals, an L = 3 is possible if the species phylogeny were to show four clades corresponding exactly to the four breeding patches, with three of the breeding patches derived from the fourth each by single events (cf. Fig 7A).

**Fig 8:**
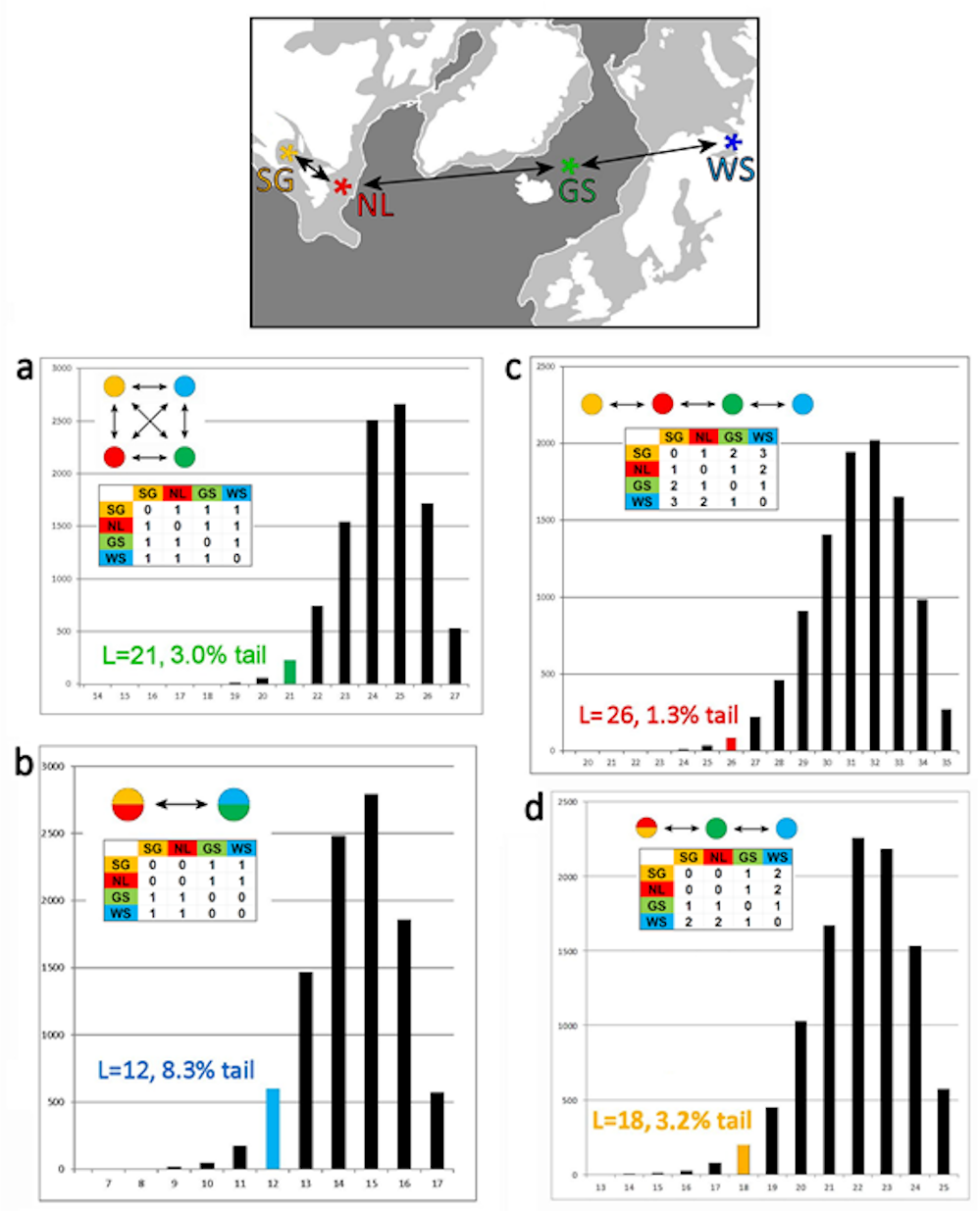
Results of Monte Carlo simulations of genomic phylogeography. Diagrams and matrices show the four main models graphically: matrix elements are the cost of dispersal events required by the tree. (a) Two-population model, SG+NL versus GS+WS; (b) Island model, equal cost for any movement among populations; (c Four-population east / west linear stepping-stone model; (d) Three-population linear stepping stone model, SG+NL combined. Summary results for these and four differentially weighted models are given in Table 4. The range of each graph includes the observed maximum length and one length class lower than the observed lower minimum.

For the Monte Carlo dispersal analysis, models and matrices for each of four main models as shown graphically in Fig 8, and summary data for these and four differentially-weighted models are given in Table 4. The range of each graph includes the maximum possible length and one length class lower than the observed lower minimum. A random distribution among breeding patches is rejected in favor of a simple island model (Fig 8, Model A: 3.0% tail). A random distribution is also rejected in favor of linear, East ◄► West trans-Atlantic stepping-stone models with two or three steps (Models D and C, 3.2% and 1.3% tails, respectively), though not for a simple one-step model (B, 8.3%). (Replicate randomizations give essentially the same results). Note that Model A is essentially equivalent to AMOVA Model I (among population **ϕ**_ST_ = 0.0580, p << 0.01). The one-step Trans-Atlantic Model B is roughly equivalent to the group partition in AMOVA Model II (ϕ_CT_ = 0.0057, p >> 0.05), except that Model B pools seals within the NWA and NEA groups, whereas Model II separates them within groups.

**Table 4:** Results of Monte Carlo simulation of alternative phylogeographic models of Harp Seal dispersal. Abbreviations as in Table 2. For each model in Fig 8, the table gives the dispersal cost (length L) required by the topology of the observed tree (Fig 2), the frequency of that L class among 10,000 randomizations, the frequency of the tail of the distribution that includes that class, and the maximum, mode, and observed minimum among the randomizations.

Model	Description	L	L freq	L-inclusive freq	Random Min	Random Mode	Random Max
A	Island	21	2.25%	*2*.*97%* *	17	25	27
B	Trans-Atlantic, WS/GS—NL/SG	12	5.99%	8.30%	8	15	17
C	Stepping stone, WS—GS—NL—SG	26	0.84%	*1*.*34%* *	21	32	35
D	Stepping stone, WS—GS—NL/SG	18	2.07%	*3*.*16%* *	14	22	25

* = p < 0.05.

**Fig 9:**
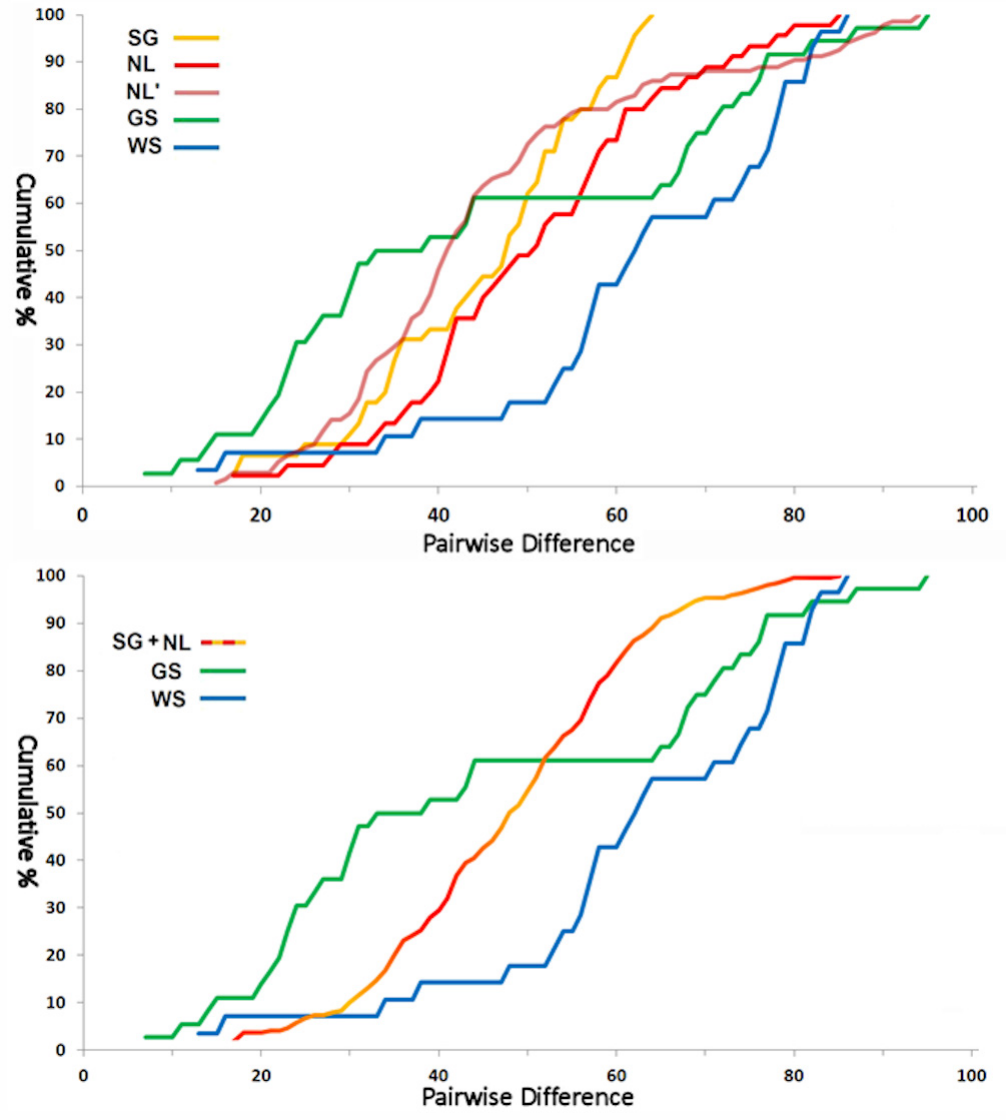
Cumulative pairwise distribution curves for the Kolmogorov-Smirnov test. (a) Curves for five samples analyzed separately; abbreviations as in Fig 8(B) Curves for the summary three-population model: SG+NL (not including replicate NL’ samples) versus GS versus WS. Note that the former is not the simple sum of the curves for the component samples, as each includes between-sample distances as well.

Results of the non-parametric Kolmogorov-Smirnov (K-S) test are given in Fig 9 and Table 5. The cumulative curves for the GS and WS samples are significantly offset (***D*** = 0.522, p << 0.01), whereas those for the NL and SG samples are not (***D*** = 0.200, p > 0.05), although those for the replicate Front sample and SG are (***D*** = 0.259, p < 0.05). The curve for the NL+GS samples combined (Table 6) is significantly differentiated from all other samples, including the replicate Front sample (***D*** = 0.211, p < 0.01). All four trans-Atlantic comparisons are significant. The same comparisons are significant, with slightly reduced p values, if ***D***_***a***_ is calculated from *(n-1)(n-2)/2*. None are significant if ***D***_***a***_ is calculated from *n*.

**Table 5:** Kolmogorov-Smirnov tests of differences among cumulative pairwise distributions. ***D*** values for pairwise comparisons among four breeding populations. ***D*** expresses the maximum difference between distributions weighted for sample size [60]. Significance was evaluated with the critical value ***D***_***a***_ calculated by two alternative criteria, either the total number of pairwise comparisons in each samples ((*n*)(*n*-1)/2), or the sample size of each samples (*n*) (see Materials and Methods). The table gives the value of ***D*** and its significance based on the first criterion. None of the ***D*** values were significant by the second criterion.

	SG	NL	GS	WS
**SG**	-	ns	**	***
**NL**	0.200	-	***	*
**GS**	0.389	0.389	-	**
**WS**	0.533	0.377	0.522	-

* = p < 0.05

** = p < 0.01

*** = p < 0.001.

**Table 6:** Kolmogorov-Smirnov tests of differences among cumulative pairwise distribution. ***D*** values for pairwise comparisons, with SG and NL samples combined. See Table 5 for considerations of ***D***, ***D***_***a***_, and *n*.

**SG+NL**	**NL'**	0.211	**
**SG+NL**	**GS**	0.356	***
**SG+NL**	**WS**	0.437	***
**SG+NL**	**GS+WS**	0.335	***

* = p < 0.05

** = p < 0.01

*** = p < 0.001.

## Discussion

The AMOVA models identify small but significant structures among the four breeding grounds *within* 2-, 3-, or 4-island models, but not *among* these model groups. In contrast, the Monte Carlo simulation and K-S tests taken together support a non-random distribution of seals, across stepping-stone as well as among island phylogeographic models in which the breeding areas comprise three distinct populations. These correspond to separate populations in the White Sea and Greenland Sea, each with distinctive phylogeographic and temporal structures, and a more homogeneous population in the Northwest Atlantic with intermediate but variable temporal structure. This is the first demonstration of genetic differentiation between the two breeding grounds in the Northeast Atlantic.

### Symplesiomorphy of cytochrome B single-locus haplotypes

Analysis of a 307bp segment of the mtDNA cytochrome *b* gene identified a common haplotype “***A***” in four out of ten seals from each of the two Northeast Atlantic areas and eight out of ten from each of the two Northwest Atlantic areas [22]. A row by column frequency test indicated significant differentiation of the Northwest and Northeast Atlantic pairs.

In Atlantic Cod (*Gadus morhua*), inspection of whole-genome sequences of the most common haplotype for the same gene region was a symplesiomorphic assemblage of unrelated lineages from different distantly related clades [4]. Here, inspection of the whole-genome sequences of the seals with the modal Cytochrome b haplotype in [22] also identifies it as a symplesiomorphic, paraphyletic assemblage that arose with the **E** clade and comprises seals from the **D**, **C**, **B** (both seals) and **A** clades as well. We previously suggested that focal genotypes in many other ‘star phylogenies’ [28] based on relatively short DNA sequences and hypothesized as indicative of recent origins may in fact be symplesiomorphic groups in much older phylogenies, which would be evident in whole-mtDNA genome analyses.

### Rates of mitogenomic evolution in *Pagophilus*

In the absence of a suitably recent biological or geological reference point for calibration of an intraspecific molecular clock in *Pagophilus* or for other phocid seals over the appropriate genetic divergences and (or) time range [29], we constructed a whole-mtDNA-genome molecular clock based on sequence data from three extant human lineages along with those of Neanderthal and a Denisovan. Sequence differences among the hominine lineages span the range observed in *Pagophilus*, and the lineage divergence times are relatively well dated though highly debated. The best linear fit has a slope of 4.5 Kyr / substitution, as compared with coding-region rates in *Homo* of 5.1 Kyr / substitution [29], a linearized Hominoid rate of 6.1 Kyr / substitution (Fig 5), and 5.7 Kyr / substitution for coding-regions in *Gadus* spp. codfish [4]. Part of the variation may be explained by use of slightly different partitions and (or) regions of homology within the coding region [cf. 30]. Other calibrations suggest that phocid mitogenomes may evolve more slowly than those of hominids [31]. Extrapolation of this curve (and variants) to date the MRCA of *Pagophilus* and its closest relative, the endemic Pacific ribbon seal (*Histriophoca fasciata*) gives a date of 3.7 ~ 4.1 MYA, which slightly antedates the most recent opening of the Bering Strait 3.5 MYA [32]. This geological event was used to calibrate the intra- [4] and interspecific [33] clocks for *Gadus* spp. The Strait has opened and closed several times over the Pleistocene [32], and the *Pagophilus* x *Histriophoca* divergence may date to one of those earlier episodes.

### Temporal implications of pairwise mismatch analysis of population expansion

Genetic biodiversity in *Pagophilus* is dominated by clade **A**, which originated and diversified as part of a population expansion 53 KYA (Figs 4B and 5). The estimated population expansion time immediately precedes the coalescent times of all five subclades in clade **A**, along with three of the other four major clades. Sequence diversity thus accompanies lineage diversification within the currently predominant lineages. The whole-genome data indicate a deep phylogeographic structure, in which the age of the deepest within-species coalescent **F** is similar to that of the exclusively African **L** lineages in *Homo*. The **F1** and **F2** lineages have been separate almost as long as the species coalescence. The exceptional seal from the replicate NL’ sample in **F1** is well separated from its closest relative, as evidence of ancient temporal divergence rather than recent dispersal, similar to what was observed in isolated populations of *Gadus chalcogrammus* [5].

### Implications of AMOVA and Monte Carlo biogeographic models

The outstanding biogeographic question in Harp Seals is the extent of movement among breeding and whelping areas, and in particular the question of whether seals from the White Sea and Greenland Sea areas are distinguishable populations. The degree of separation of the Front and Gulf populations also affects questions of management of numbers in those two areas [34,35].

The observed phylogeographic distribution is significantly structured with respect to random expectation (a simple ‘island’ model). All stepping-stone models fall within the shortest 5% tail of all distributions, except for the one-step, two-population Model B. The models that depart mostly strongly from random are the four- and three-step trans-Atlantic stepping-stones, where the observed distribution falls within the 1.3% and 2.1% tails, respectively. The four-island AMOVA Model I also identifies significant ϕ_ST_ among populations. Thus the phylogeographic distribution is significantly structured, though the significance of the smaller number of dispersal events L with respect to the random distribution as measured by the p-value ranking of island *versus* two- or three-step stepping-stone models cannot be tested at present. The rankings of stepping-stone models seem to be dominated by the marked differentiation of WS and GS, as is also evident in the K-S tests, below. Differentiation of WS from other breeding grounds is also shown by significant pairwise ϕ_ST_ and suggested by AMOVA Model IV, though the ϕ_CT_ is non-significant.

### Implications of cumulative pair-wise mismatch for phylogeographic structure

The program DnaSP (see Methods) uses the distribution of pairwise mismatches among individuals within species to test whether the species showed evidence of constant size or expansion, and if so when. Prompted by this approach, we developed a novel analysis that uses a cumulative pair-wise mismatch curve calculated for each population segment as a proxy for its branching phylogenetic structure. Differences between the structures of any two populations can then be evaluated quantitatively by a non-parametric Kolmogorov-Smirnov (K-S) test of the maximum vertical offset between curves (see Methods). Here, K-S identifies the greatest disparity in the time required to reach a comparable level of phylogenetic diversity. The position of this disparity on the horizontal (cumulative pairwise difference) axis is heuristically valuable, as is the relative ranking of curves at 50% cumulative pairwise differences.

In Fig 9A, the significant vertical offset of curves for the GS and WS samples reflects the different phylogenetic histories of the two populations. As noted in Figs 2 and 3, seals from WS occur almost exclusively in the basal clades, so that the modal pairwise difference is larger and the curve shifted rightward (>50% of differences >60). Seals from GS occur predominantly in the relatively younger **A** clade, such that the modal difference is smaller and the curved shifted leftward (>50% of differences < 40), with the exception of the basal subclade **F1**, which shifts the last ~40% of differences closer to the East curve. The structure of GS (allowing for small sample size) is consistent with an older population with a relatively recent bottleneck in numbers such that the numbers of the minority clades **B**, **C**, **D** & **E** have been severely diminished, and only the plurality **A** clade survives alongside the basal **F1**. The historical catch records indicate that the population was severely depleted by the 1870s and remained low until the 1960s when it began to recover [11]. In contrast, the basal mtDNA haplotype composition as quantified by the displacement of the cumulative curve to the right indicates a much older population. Finally, lack of significant differentiation between the cumulative curves for NL and GS (though the latter is consistently to the left of the former) suggests similar population dynamics in the two, consistent with their treatment as a single biological entity.

Comparison of relative ranks among populations across the horizontal pairwise difference axis clarifies the phylogeography of the replicate Front samples. Differences among the four main samples are consistently ranked GS < SG < NL < WS, but the replicate NL (NL’) curve crosses the others at several points. Inspection of Fig 2 suggests a broad qualitative similarity between the phylogenetic distributions of NL’ and GS samples, *i*.*e*., almost all component seals are in the **A** clade, with outliers in the more basal clades. However, the NL’ and GS curves are distinct, the former with a cumulative 50% reached at 42 differences, and the latter with a cumulative 50% at only 33 differences, and the two curves crossing at ~55% / 45 differences. This seems to indicate, within the **A** clade, a more recent modal relationship among GS seals than among NL’ seals. The Front curve also crosses the Gulf curve at 30% and again at 80%: the latter seems to reflect its inclusion of basal individuals that are absent in the Front and Gulf samples. Fig 9B summarizes the three-population model as a combined NL+SG curve for comparison with the separate GS and WS curves from Fig 9A. The combined NWA curve is clearly distinct from both of the latter, and crosses the GS curve at about 60% at ~ 50 differences. Thus, the method of cumulative mismatch curves as applied to intra-specific genomic trees seems to be sensitive to subtle differences in phylogeographic structure of populations of mixed phylogenetic relationship, even with relatively small sample sizes. A limitation of K-S is that only the maximum ***D*** is evaluated, although this maximum is typically part of a continuous trend in cumulative differences: it would be useful to identify significant ranges.

### Implications for Harp Seal ecology and management

An understanding of the relationships among the various Harp Seal whelping areas is critical for proper science-based management during the present period of climate change in the North Atlantic [36,37,38,39,40]. Allowable catches are based upon abundance estimates derived primarily from pup production surveys in each of the whelping areas. Pup production in the White Sea declined unaccountably by more than 50% between 2003 and 2006 [41]. This decline has in part been attributed to changes in maternal body condition [42], but may also be a consequence of the movement of seals from their traditional whelping locations. Harp seals whelp along the southern limits of pack ice: as ice conditions in the North Atlantic continue to deteriorate due to climate change [43,44,45,46], it is likely that seals will be forced to move to new areas to give birth. In 2010, seals in the Northwest Atlantic were observed whelping north of their traditional area, which was attributed to a lack of suitable ice for pupping [47]. Similarly, seals have recently been reported whelping on pack ice off Cape Farewell at the southern tip of Greenland [48], an area of overlap between seals from the Northwest Atlantic and Greenland Sea in the non-whelping hunting season [49], but with no historical records of pupping.

Currently, the Northwest Atlantic is managed as a single population with quotas allocated between the Front and Gulf based upon the relative historical number of pups in each area. However, is some years, very little pupping occurs in the southern Gulf and Gulf animals move northward where they may mix with animals that normally breed at the Front [11,47]. A pattern of general patch fidelity in combination with inter-annual variation in degrees of intermixing may explain the significant differentiation of the replicate Front and SG samples, and the degree of departure from random of the three- and four-population models, with NL and SG combined or not. As ice conditions in the Gulf of St Lawrence continue to deteriorate, demographic and genetic relationship between the two Northwest Atlantic sub-populations may change, so as to require a change in the management approach [47].

### Opportunities and challenges

Whole-mitogenome sequences offer fully-resolved phylogenetic trees within species that are literal “family trees” for maternal lineages. This raises new opportunities and challenges for data analysis. Assessments that differences among phylogenetic trees drawn from different populations or geographic segments are ‘different’ is elusive, not least because of the multiple ways in which such trees may be ‘different’. AMOVA partitions nucleotide variance but does not capture phylogenetic structure. We address two aspects of differentiation, first whether phylogeographic distribution is structured or random, and second whether temporal phylogenetic patterns among populations are distinguishable. Harp Seals are an ideal species in which to test ideas about biogeography and intraspecific mitogenomic phylogeny, because the discrete geographic distribution among a limited number of breeding areas provides several *a priori* hypotheses. A novel Monte Carlo method shows that different biogeographic models have greater or lesser departures from random expectation, and thus represent stronger or weaker hypotheses of structure. In other, more continuously distributed species, mitogenomic data offer the potential for population structures to be detected in more dispersed collections of individual genome-types. Our non-parametric analysis assesses cumulative mismatch curves as proxies for intra-specific genomic trees, by converting a complex branching topology to a continuous curve approximation of phylogenetic diversity over time. Although the K-S ***D*** statistic assesses the single maximum difference between curves, the maxima seen here are typically the high point on trending differences between curves. The method is shown here to be sensitive to subtle differences in temporal structure of populations of mixed phylogenetic composition.

## Materials & Methods

### Samples and DNA sequencing

Seal DNA samples include 37 out of 40 of those analyzed in [22] from the three breeding areas, including separate samples from the Newfoundland and Labrador coastal Front and the southern Gulf of St Lawrence, plus 16 new samples from the Front. All tissue samples were collected from seals taken on the respective whelping areas under scientific permits issued by the Norwegian and Russian governments, and by Department of Fisheries and Oceans scientists under permission granted by Section 52 of the Fisheries Act of Canada. All seals were killed according to the humane killing methods legally required in the respective countries, and were taken as part of ongoing research on population dynamics. No animals were killed specifically for the present study. DNA was also extracted from samples obtained by punch biopsy from the hind flippers of 16 additional seals on the Front in 1994 and 1999, by DFO scientists, under the terms and conditions of the Fisheries Act.

Complete 15,825 bp mitogenomes (without the D-Loop Control Region) were generated by the polymerase chain reaction with a set of 20 primer pairs that amplify the mitochondrial genome in fragments of 750~1400 bp, with overlaps between adjacent fragments of 80~300 bp. Of the 53 genomes, 45 were sequenced on a custom-designed multi-species iterative re-sequencing microarray, the “ArkChip” (Affymetrix) [50]. Our base-calling algorithm integrates differential fluorescence intensities of base-specific oligonucleotide binding to bases on either DNA strand with empirical rules for identification of SNPs [51]. The remaining eight genomes were sequenced with the BigDye chemistry v.2.0 (Applied Biosystems) on an ABI377 Prism automated sequencer. The entire set of 53 sequences was assembled with the Sequencher 4.9 program (GeneCodes, Ann Arbor MI). Both DNA strands were sequenced for all individuals.

### Phylogenetic analysis and calibration of the molecular clock

For the genomic data, a neighbor-joining (NJ) tree was identified from the absolute number of nucleotide differences (10,000 bootstrap replications) (MEGA5 [52]). A maximum parsimony (MP) tree was identified by heuristic search of equally-weighted SNP differences among haplotypes (TBR branching swapping and 100 taxon additions for identification of the minimum tree, 10,000 bootstrap replications with SPR swapping and 5 additions each) (PAUP*, v.4.10 [53]). A maximum likelihood (ML) tree from a general time-reversible model was used to correct substitution rates for calculation of absolute times to MRCAs either by clock-constrained (PAUP*) or linearized (MEGA5) models. A Bayes tree was calculated with the GTR substitution model and gamma-distributed rate variation over sites with allowance for invariable sites. A total of 2,000,000 generations were run, with a final standard deviation of the split frequencies <0.008 (MrBayes, v.3.2 [54]). Pairwise mismatches and tests of the mismatch distribution were calculated with DnaSP v.5.1.10 [55].

In the absence of any well-defined recent biological or geological reference point for calibration of an intra-specific *Pagophilus* molecular clock [25], we estimated a clock-constrained maximum likelihood model by means of four hominines with complete mtDNA genome sequences and known MRCAs in the interval 70 ~ 1,000 KYA with respect to the senior author’s mitogenome in Haplogroup **U** of macrohaplogroup **N**. These are Fr2 in Haplogroup **A1** of macrohaplogroup **M** [9], San2 in Haplogroup **L1** [1], a Neandertal [56], and a Denisovan [57]. The calibration was calculated for a 15,383 bp region homologous with the *Pagophilus* mitogenome alignment.

### Analysis of molecular variance

We used Arlequin v.3.5 [58] to calculate all pairwise ϕ_*ST*_ values among populations based on nucleotide distances among haplotypes. We tested three partitions of within- and among-group variance (ϕ_SC_ and ϕ_CT_, respectively) [59], corresponding to two-, three-, and four-island models of Harp Seal structure based on *a priori* east-to-west groupings of breeding grounds. We also tested a fourth *a posteriori* two-island model with the eastern-most White Sea breeding ground separated from the other three, based on the observation of significant ϕ_ST_ between WS *versus* NL, and *versus* GS.

### Phylogeographic analysis by Monte Carlo simulation

We developed two new analytical methods and applied to the intraspecific phylogeographic tree. We first sought to determine how well the observed phylogeography fit various *a priori* models suggested by the geographic distribution of breeding populations, relative to the random distribution expected for such models. We performed a series of Monte Carlo randomizations of population assignments over the tree topology in Fig 2, as follows. We first used an Excel spreadsheet to generate 10,000 sets of 53 random numbers. For each set, we coded each number according to its rank in the set into a new matrix as a character state a, g, c, or t, in successive blocks with the same proportions as the observed population assignments (SG = a = 10, NL = g = 26, GS = c = 9, & WS = t = 8). Use of a, g, c, & t as character states allows the computationally efficient datatype = dna option of PAUP to be used. The resulting 53 x 10,000 matrix then corresponds to a PAUP file where each of 10,000 characters is a tree with the observed topology, but with population assignments randomized over the 53 branch tips. Given the topology of the observed tree (Fig 2) as a treefile, we next use PAUP to ascertain the number of dispersal events (**L**) required to explain the observed tree, *versus* each of the random assignments over that same topology. We finally ask where **L** of the observed tree falls relative to the distribution of **L** over the random assignments. Different phylogeographic models can be represented encoded as a set of 4x4 Character Type matrices that count the stepwise cost of dispersal events among populations.

We tested four models (Table 4): (a) an island model with unit cost for any event between populations [unordered character matrix], (b) a two-population model with a unit cost only for a trans-Atlantic event, (c) a four-population east-west stepping-stone model with unit cost for each step in the series SG ◄► NL ◄► GS ◄► WS, and (d) a three-population variant of Model (c), with a cost of 0 for an SG ◄► NL event. All matrices were symmetrical (bidirectional dispersal). Diagrams and matrices for each of the models are shown graphically in Fig 8.

### Non-parametric analysis of phylogenetic structure

Differences between population-specific cumulative pairwise difference curves were evaluated with a non-parametric, two-sample Kolmogorov-Smirnov (K-S) test procedure [60]. For a continuous two-population comparison, the method identifies the largest unsigned observed difference (***D***) between the cumulative frequency distribution over the range of observed differences. Here, the cumulative curves are the cumulative numbers of pairwise differences in each sample, and ***D*** is the vertical offset between curves. The significance of ***D*** is evaluated by the critical value ***D***_***a***_, which calculation includes counts of the number of comparisons within each population as a measure of the degrees of freedom. For pairwise data this would ordinarily be (*n*)(*n*-1)/2, however because the contributions of each individual to the set of pairwise difference comparisons are not independent, ***D***_***a***_ may more accurately be estimated from the total number of individuals *n* within both populations (J. Rohlf, pers. comm.) or by (*n*-1)(*n’*-1)/2 for an n x n’ matrix. We evaluated ***D*** on all three criteria.

## Supporting Information

S1 Table Pairwise differences among mtDNA genomes of 53 Harp Seals (*Pagophilus groenlandicus*). (DOCX)
