# Supplementary figures and images for: Quantitative Phylogenomics of Within-Species Mitogenome Variation: Monte Carlo and Non-Parametric Analysis of Phylogeographic Structure among Discrete Transatlantic Breeding Areas of Harp Seals (Pagophilus groenlandicus)
Source: PLoS One. 2015 Aug 24;10(8):e0134207. doi: 10.1371/journal.pone.0134207 (PMC4547794; doi:10.1371/journal.pone.0134207)

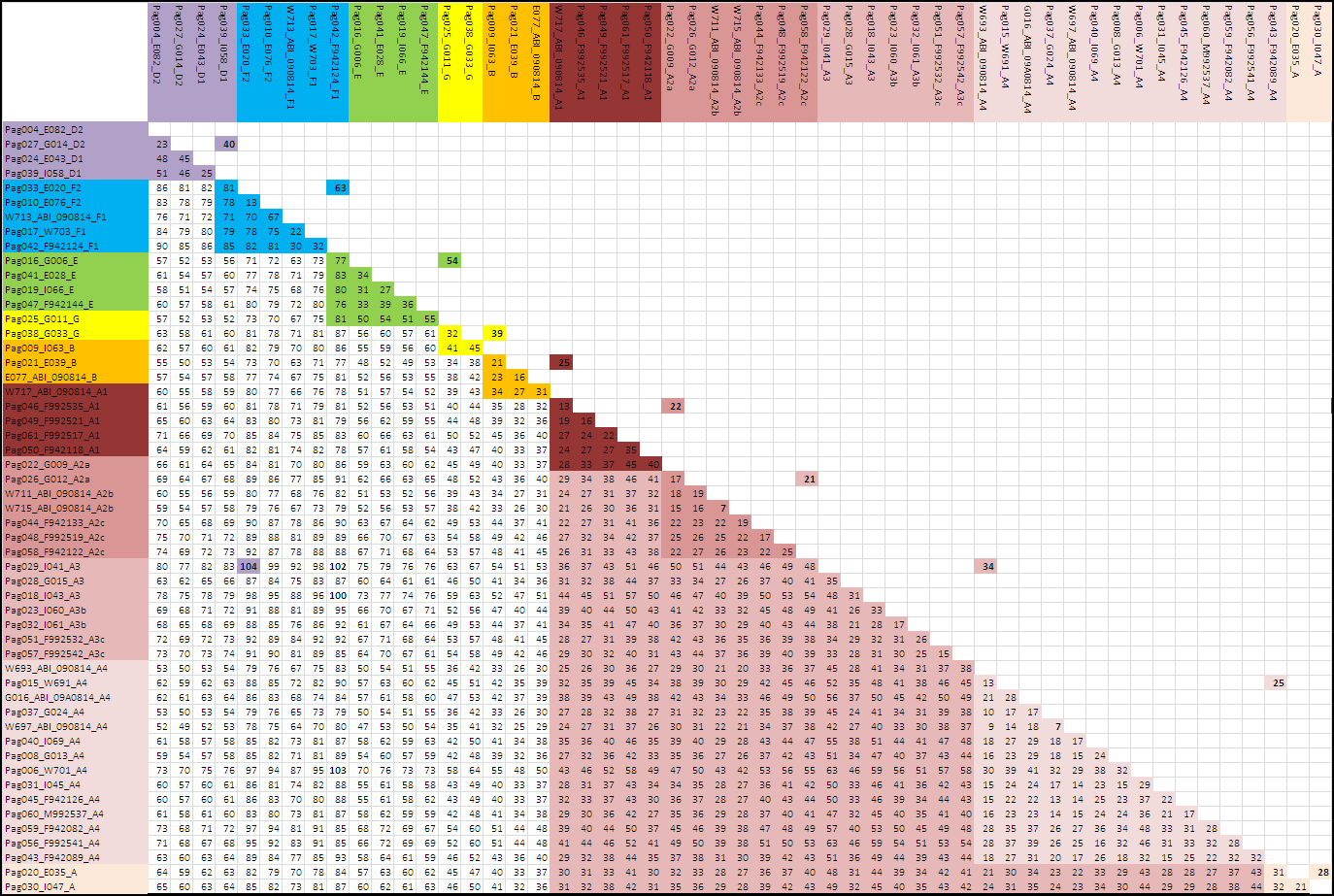
Table S1 – Pairwise differences among mtDNA genomes of 53 Harp Seals (*Pagophilus groenlandicus*)

Supplement: S1 Table — (DOCX) [file pone.0134207.s001.docx]
